# Quantitative thermal testing as a screening and follow-up tool for diabetic sensorimotor polyneuropathy in patients with type 2 diabetes and prediabetes

**DOI:** 10.3389/fnins.2023.1115242

**Published:** 2023-03-27

**Authors:** Yu-Chuan Huang, Yao-Chung Chuang, Wen-Chan Chiu, Chih-Cheng Huang, Ben-Chung Cheng, Chun-En Aurea Kuo, Ting-Yin Lin, Hui-Ching Chiang, Yun-Ru Lai

**Affiliations:** ^1^Department of Neurology, Kaohsiung Chang Gung Memorial Hospital, Chang Gung University College of Medicine, Kaohsiung, Taiwan; ^2^Department of Neurology, Pao Chien Hospital, Pingtung, Taiwan; ^3^Department of Neurology, School of Medicine, College of Medicine, Kaohsiung Medical University Hospital, Kaohsiung Medical University, Kaohsiung, Taiwan; ^4^Department of Biological Science, National Sun Yat-sen University, Kaohsiung, Taiwan; ^5^Department of Internal Medicine, Kaohsiung Chang Gung Memorial Hospital, Chang Gung University College of Medicine, Kaohsiung, Taiwan; ^6^Department of Chinese Medicine, Kaohsiung Chang Gung Memorial Hospital, Chang Gung University College of Medicine, Kaohsiung, Taiwan; ^7^Department of Nursing, Kaohsiung Chang Gung Memorial Hospital, Chang Gung University College of Medicine, Kaohsiung, Taiwan; ^8^Hyperbaric Oxygen Therapy Center, Kaohsiung Chang Gung Memorial Hospital, Chang Gung University College of Medicine, Kaohsiung, Taiwan; ^9^Department of Neurology, Xiamen Chang Gung Memorial Hospital, Xiamen, Fujian, China

**Keywords:** diabetic sensorimotor polyneuropathy (DSPN), prediabetes, quantitative thermal testing (QTT), Toronto Clinical Neuropathy Score, type 2 diabetes mellitus, nerve conduction study (NCS)

## Abstract

**Introduction:**

The diagnosis and assessment of neuropathy severity of diabetic sensorimotor polyneuropathy (DSPN) are mainly based on clinical neuropathy scores and electrophysiologic studies. This study aimed to determine whether quantitative thermal testing (QTT) can be used as a screening and follow-up tool for DSPN of prediabetes and type 2 diabetes at baseline and at 1-year follow-up.

**Methods:**

All patients were assessed using the Toronto Clinical Neuropathy Score (TCNS) and underwent electrophysiological testing, including a nerve conduction study (NCS) and QTT, at baseline and at a 1-year follow-up. The TCNS and the composite scores of nerve conduction were used to assess the severity of DSPN. The DSPN status at the 1-year follow-up was classified as remaining no DSPN, remaining DSPN, regression to no DSPN, or progression to DSPN.

**Results:**

Diabetic sensorimotor polyneuropathy was initially diagnosed in 89 patients with prediabetes and type 2 diabetes (22%). The regressed to no DSPN in 29 patients and progressed to DSPN in 20 patients at the 1-year follow-up. TCNS was significantly correlated with composite scores of nerve conduction, hand cold detection threshold (CDT), hand warm detection threshold (WDT), foot CDT, and foot WDT. Stepwise logistic regression demonstrated that the foot CDT (*p* < 0.0001) was independently associated with the presence of DSPN. The TCNS, composite scores of the nerve conduction, hand WDT, hand CDT, foot WDT, and foot CDT were all statistically significant among the four different DSPN status groups at two different time periods (baseline and the 1-year follow-up).

**Conclusion:**

The foot CDT can be used as an initial screening tool for DSPN alternatively. The characteristics of nerve damage after 1 year of DSPN can be progressive or reversible, and the neurological functions of large and small fibers have a parallel trend, which can be objectively measured by NCS and QTT.

## Introduction

Diabetic sensorimotor polyneuropathy (DSPN), a major cause of foot ulcers and amputation, is also a major microvascular complication of type 2 diabetes mellitus (T2DM) (Ziegler et al., 2014). The current level of evidence confirmed that DSPN could develop in parallel and may be progressive and reversible (Ziegler et al., 2021). Recently, accumulating pieces of evidence demonstrate that subjects with impaired fasting glucose and impaired glucose tolerance could increase the prevalence rate in DSPN (Ziegler et al., 2014). The American Diabetes Association position statement recommends neurologic screening for prediabetic patients in addition to diabetic patients complaining of symptoms of peripheral neuropathy (Feldman et al., 2019).

Previous studies have shown a high unawareness prevalence of clinical DSPN in prediabetic and diabetic patients (Ziegler et al., 2014). DSPN can involve small fibers, large fibers, or both, and it is considered the most common type of peripheral neuropathy in both type 2 diabetes mellitus (T2DM) and prediabetes (Stirban, 2015). In peripheral nerves, C-type nerve fibers are involved in the perception of thermal stimuli, while Aδ nerve fibers are cold stimuli. Although the quantifying intraepidermal nerve fiber density from a skin biopsy has confirmed the standard for diagnosing small fiber neuropathy in diabetes (Feldman et al., 2019), quantitative thermal testing (QTT) is considered a reliable tool for the diagnosis of somatic small fiber neuropathy in clinical practice and also be used for monitoring progression in follow-up studies (Dyck et al., 1978; Shy et al., 2003; Chong and Cros, 2004) because it is quantifiable and reproducible.

Our study aimed to determine whether QTT could be used as a time-saving surrogate tool to identify prediabetes and type 2 diabetes patients at higher risk of DSPN in outpatient settings, and to analyze which QTT parameters could serve as indicators for follow-up DSPN. The results of this study are expected to provide clinicians with more references to provide or refer DSPN patients with diabetes and prediabetes for effective preventive and therapeutic interventions.

## Materials and methods

### Study subjects

**FIGURE 1:**
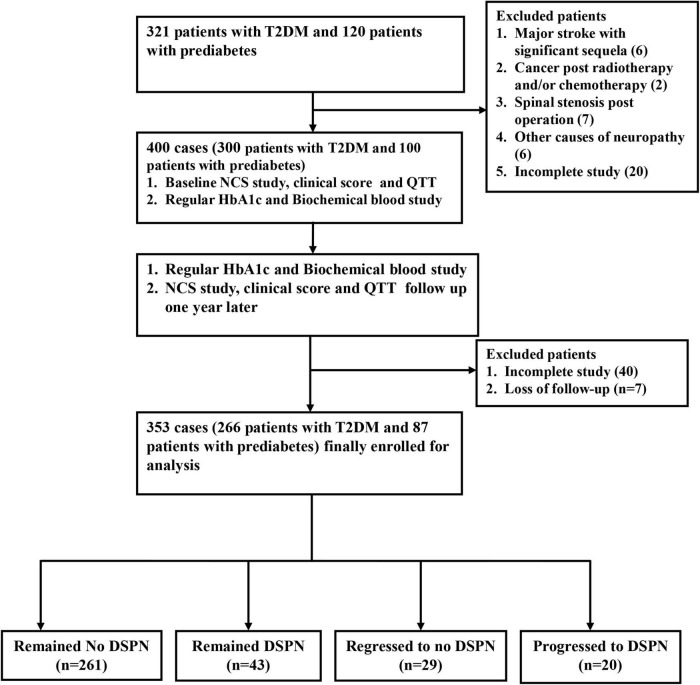
Flow diagram of the patient selection method.

The single-center, prospective, case-control study was conducted at a tertiary medical center and main referral hospital in southern Taiwan. The baseline survey and follow-up survey since 2019–2021 included 321 type 2 diabetic patients and 120 patients with prediabetes. Prediabetes is defined as having elevated blood sugar levels and hemoglobin A1C levels of 5.7–6.4% (American Diabetes Association, 2014). DSPN was defined according to the Toronto Consensus on diabetic neuropathy, which was confirmed as patients who had neurological symptoms and/or signs and abnormalities of NCS (England et al., 2005; Tesfaye et al., 2010). Subjects with other causes of neuropathy (except for diabetes/prediabetes) or serious cognitive impairment were excluded based on detailed history and blood tests. A total of 400 patients (300 with T2DM and 100 with prediabetes) were enrolled in this study (Figure 1). This study was approved by the review committee on human research of the hospital (201901363B0 and 202002095B0). All participants received verbal and written information about the purpose and process of the study and provided informed consent.

### Baseline clinical and laboratory measurements

All patients underwent complete neurological and physical examinations at enrollment and subsequent follow-up sessions in the outpatient clinic by an experienced neurologist (Yun-Ru Lai). A complete history was obtained for each participant, and the data collected included the age at the disease onset, sex, height, weight, body mass index (BMI), disease duration, systolic blood pressure, diastolic blood pressure, and microvascular complications of diabetes. Laboratory parameters were obtained after enrollment using standardized questions. For each patient, the estimated glomerular filtration rate (eGFR) (Ma et al., 2006) and urinary albumin-to-creatinine ratio (UACR) was measured as shown in previous studies (Chiu et al., 2020), and the intrapersonal mean and standard deviation (SD) of the HbA1c were calculated using all measurements obtained 3 years before the beginning of the study (Kilpatrick et al., 2007; Lai et al., 2019).

### Quantitative thermal testing and Toronto Clinical Neuropathy Score

The QTT values for the thermal perception thresholds were measured using the sensory threshold evaluation system (Q-Sense Thermal Sensory Analyzer, Medoc Advanced Medical Systems, Ramat Yishai, Israel) according to established protocols as follows (Yarnitsky and Sprecher, 1994; Chao et al., 2007; Huang et al., 2010). All participants were examined to experience a warm sensation and were asked to maintain a high level of concentration, but no further instruction or training was provided. The temperature was increased to detect the warm threshold and decreased to detect the cold threshold. A validated questionnaire, the TCNS, was completed for all participants. Six points are from symptoms, eight points from lower limb reflexes, and five points from the sensory examination. It is a continuously variable range reflecting the severity of DSPN on a scale from 0 to 19 (Bril and Perkins, 2002).

### Nerve conduction studies

Nerve conduction study was performed by a trained electrophysiologist using Nicolet Viking machines (Madison, WI, USA). We performed motor nerve studies, including the median, ulnar, tibial, and peroneal nerves, and sensory nerve studies including the median, ulnar and sural nerves. The parameters measured for each nerve included distal latency, amplitude, and nerve conduction velocity, and all data obtained were compared with reference values from our laboratory (Huang et al., 2009). The sensory and motor nerves on the bilateral side were tested, and only the nerves on the non-dominant side were recorded in the DSPN.

To improve the assessment of diabetic neuropathy using nerve conduction studies, Dyck et al. (1997) constructed composite scores of nerve conduction to evaluate the severity of DSPN. The composite score of nerve conduction was composed of the peroneal compound muscle action potential (CMAP), tibial CMAP, ulnar CMAP, sural sensory nerve action potential (SNAP), and ulnar SNAP amplitudes (Dyck et al., 2019). These percentile values were expressed as points from the percentile values obtained according to our previously published study, and the five attributes of the NCS provided a scale from 0 to 10 points (Dyck et al., 2019; Lai et al., 2019).

### Outcome assessment

Patients were classified into four groups according to the DSPN status over the 1-year follow-up as follows: no DSPN, remaining DSPN, regression to no DSPN, and progression to DSPN (Ziegler et al., 2021).

### Statistical analysis

The categorical variables were compared using chi-squared or Fisher’s exact tests. The continuous variables are expressed as the mean ± SD or as the median (interquartile range). Variables that were not normally distributed were logarithmically transformed before the analysis to improve the normality. The continuous variables were compared between the two groups using an independent *t*-test. Correlation analysis was performed to evaluate the relationship between the composite scores of the nerve conduction, TCNS, and QTT parameters. Continue variable and categorial variables at baseline and 1-year follow-up in T2DM and pre-diabetes were compared by Paired t test and McNemar test, respectively. The effects of the individual variables, including the baseline characteristics, underlying conditions, biochemical data, and QTT parameters on the presentation of DSPN, were analyzed using univariate stepwise logistic regression. Stepwise logistic regression, with adjustments for potential confounding factors, was used to evaluate the relationship between significant variables and the presence of DSPN. Only variables with a strong association with DSPN (*p* < 0.05) were included in the stepwise logistic regression model. Sensitivity analysis using E-values was to evaluate how strongly an unmeasured confounder would have to be related to the treatment and outcome to explain away the observed association. The composite scores of the nerve conduction and QTT parameters at baseline and at the 1-year follow-up were compared between the four DSPN status groups using a paired *t*-test. The composite scores of the nerve conduction and QTT parameters among the four groups at two-time points (baseline at enrollment, and 1-year follow-up) were analyzed by repeated-measures analysis of variance (ANOVA). All statistical analyses were performed using SPSS Statistics software (v23, IBM; Redmond, WA, USA).

## Results

### General characteristics of patients

The 400 patients included 300 with T2DM and 100 with prediabetes. The patient characteristics at baseline and 1-year follow-up in T2DM and pre-diabetes are presented in Table 1. Except index HbA1C (%) in T2DM (*p* < 0.0001) and prediabetes (*p* = 0.04), and eGFR (mL/min/1.73 m^2^) in prediabetes (*p* = 0.003) showed statistical significance between baseline and 1-year follow-up. The other parameters in T2DM and prediabetes were similar between baseline and 1-year follow-up.

**TABLE 1:** Baseline characteristics and 1-year follow-up in patients with T2DM and pre-diabetes.

	T2DM (*n* = 300)			Prediabetes (*n* = 100)		
	Baseline	1 year	*P*-value	Baseline	1 year	*P*-value
**Characteristics**						
Age (years)	70.0 ± 8.2			67.7 ± 9.4		
Sex (female/male)	169/131			43/57		
Diabetes duration (years)	9.5 ± 8.8			–		
Height (cm)	161.4 ± 7.8	161.1 ± 0.5	0.19	161.2 ± 7.8	161 ± 8.5	0.30
Weight (kg)	69.7 ± 14.0	69.3 ± 13.4	0.40	65.9 ± 10.1	66.2 ± 10.3	0.24
Body mass index	26.7 ± 4.9	26.7 ± 5.1	0.93	25.3 ± 3.2	25.6 ± 3.5	
Waist circumstance (cm)	93.3 ± 10.6	93.3 ± 11.1	0.99	8.9 ± 8.9	89.3 ± 8.5	0.14
SBP (mmHg)	137.7 ± 19.0	138.2 ± 19.0	0.68	135.3 ± 18.5	134.7 ± 14.1	0.14
DBP (mmHg)	77.2 ± 12.2	76.5 ± 11.4	0.38	76.4 ± 11.9	76.1 ± 8.8	0.72
Smoking	42	34	0.55	7	5	0.17
**Underlying disease**						
Hypertension	225	190	1.0	71	61	1.0
Hyperlipidemia	158	137	0.45	64	51	1.0
**Baseline laboratory test findings**						
HbA1C (%)	7.2 ± 1.0	7.0 ± 0.8	<0.0001	6 ± 0.3	5.9 ± 0.3	0.04
Total cholesterol (mmol/L)	161 ± 27.9	156 ± 30.1	0.015	170.9 ± 27.4	171.4 ± 28	0.81
Triglycerides (mmol/L)	147.5 ± 92.0	132.3 ± 64.3	0.003	112.2 ± 50.7		
HDL-C (mmol/L)	47.3 ± 12.1	48.0 ± 12.5	0.268	53.7 ± 12.8	52.7 ± 12.7	0.11
LDL-C (mmol/L)	85.4 ± 30.2	82.8 ± 24.8	0.17	92.5 ± 26.0	96.9 ± 23.4	0.27
UA (mmol/L)	6.1 ± 1.5	6.0 ± 1.6	0.44	5.3 ± 1.2		
UACR (mg/g)	14.8 (7.2, 57.2)	19.5 (8.4, 73.8)	0.32	7.8 (5.1, 14.0)	7.8 (4.9, 13.7)	0.32
eGFR (mL/min/1.73 m^2^)	75.1 ± 28.1	75.0 ± 27.0	0.92	87.4 ± 18.8	84.9 ± 16.8	0.003

Data are presented as the mean ± standard deviation, as the number (%), or as the median (interquartile range). T2DM, type 2 diabetes mellitus; n, number of cases; SBP, systolic blood pressure; DBP, diastolic blood pressure; HDL-C, high-density lipoprotein cholesterol; LDL-C, low-density lipoprotein cholesterol; UA, uric acid; eGFR, estimated glomerular filtration rate; UACR, urine albumin-creatinine ratio.

### NCS and QTT at baseline and 1 year follow-up in patients with T2DM and pre-diabetes

Nerve conduction study and QTT at baseline and 1 year follow-up in patients with T2DM and pre-diabetes, are listed in Table 2. The peroneal CMAPs and foot WDT at baseline and 1-year follow-up in T2DM showed statistical significance (*p* = 0.02, *p* = 0.04). The peroneal and tibial MNCV and sural SNCV at baseline and 1-year follow-up in pre-diabetes showed statistical significance (*p* = 0.042, *p* = 0.02 and *p* = 0.02, respectively). The other parameters in T2DM and prediabetes were similar between baseline and 1-year follow-up.

**TABLE 2:** NCS and QTT at baseline and 1-year follow-up in patients with T2DM and pre-diabetes.

	T2DM (*n* = 300)			Prediabetes (*n* = 100)		
	Baseline	1 year	*P*-value	Baseline	1 year	*P*-value
**NCS**						
Composite scores	2.9 ± 2.7	3.1 ± 2.7	0.66	1.7 ± 1.0	1.6 ± 0.9	0.66
**Peroneal nerve, motor**						
CMAP, left (mV)	4.4 ± 2.4	4.1 ± 2.4	0.02	5.3 ± 2.2	5.2 ± 2.2	0.47
MNCV	44.5 ± 2.7	44.9 ± 5.0	0.2	48.4 ± 4.5	47.5 ± 4.8	0.04
**Tibial nerve**						
CMAP, left (mV)	10.3 ± 5.0	10.0 ± 4.9	0.13	12.1 ± 4.8	13.3 ± 13.2	0.39
MNCV	44.5 ± 5.0	44.6 ± 4.7	0.63	47.7 ± 5.3	46.5 ± 4.2	0.02
**Sural nerve, sensory**						
SNAP, left (μV)	9.2 ± 6.0	8.8 ± 5.7	0.17	11.9 ± 6	12.7 ± 7.5	0.27
SNCV	49.4 ± 6.3	49.0 ± 6.5	0.40	50.4 ± 5.4	48.8 ± 6.3	0.02
**QTT**						
Hand WDT	35.4 ± 2.1	35.5 ± 2.7	0.64	34.7 ± 1.5	35 ± 1.9	0.1
Hand CDT	29.8 ± 1.33	29.8 ± 1.6	0.84	30 ± 1.7	30 ± 0.6	0.14
Foot WDT	41.6 ± 4.0	41.0 ± 4.1	0.04	39.6 ± 4.1	40 ± 3.5	0.35
Foot CDT	27.7 ± 2.8	27.8 ± 3.4	0.96	29 ± 1.5	29.1 ± 1.8	0.68
DSPN percentage (%)	81 (27%)	57 (21.4%)	0.27	8 (8%)	6 (6.8%)	1.0
**DSPN^‡^**						
n	300	266		100	87	
Remain normal (%)	219 (73%)	185 (69.5%)		92 (92%)	76 (87.4%)	
Remained abnormal (%)	81 (27%)	41 (15.4%)		8 (8%)	2 (2.3%)	
Regressed to normal (%)	0	24 (9%)		0	5 (5.7%)	
Progressed to abnormal (%)	0	16 (6%)		0	4 (4.6%)	

Data are presented as the mean (standard deviation). n, number of cases; DSPN, diabetic sensorimotor polyneuropathy; NCS, nerve conduction study; CMAP, compound muscle action potential; SNAP, sensory nerve action potential; MNCV, motor nerve conduction velocity; SNCV, sensory nerve conduction velocity; QTT, quantitative thermal testing; CDT, cold detection threshold; WDT, warm detection threshold. ‡ = only DSPN and no NSPN at baseline.

### Correlation analysis of the composite scores of the nerve conduction and TCNS on the QTT parameters

**FIGURE 2:**
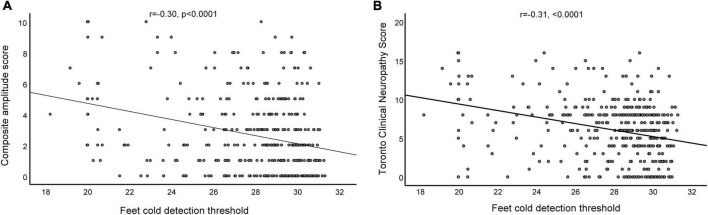
Relationship between the composite amplitude score and foot cold detection threshold **(A)**, and the relationship between the Toronto Clinical Neuropathy Score and foot cold detection threshold **(B)** in patients with diabetic sensorimotor polyneuropathy.

The parameters in the correlation analysis used to evaluate the relationship between the composite scores of the nerve conduction, TCNS, and QTT parameters are listed in Table 3. The statistically significant results (correlation coefficient, *P*-value) between composite scores of the nerve conduction and QTT parameters were as follows: hand WDT (*r* = 0.27, *p* < 0.0001), hand CDT (*r* = −0.14, *p* = 0.006), hand CDT (*r* = 0.26, *p* < 0.0001), and foot CDT (*r* = −0.30, *p* < 0.0001). Furthermore, the statistically significant results (correlation coefficient, *P*-value) between TCNS and QTT parameters were as follows: hand WDT (*r* = 0.15, *p* = 0.003), hand CDT (*r* = −0.14, *p* = 0.006), hand CDT (*r* = 0.26, *p* < 0.0001), and foot CDT (*r* = −0.31, *p* < 0.0001) (Figures 2A, B).

**TABLE 3:** Correlation analysis of the composite scores of the nerve conduction and TCNS on the QTT parameters.

Variables	Composite scores		TCNS	
	*r*	*P*-value	*r*	*P*-value
TCNS	0.521	<0.0001	–	–
Composite scores	–	–	0.521	<0.0001
**QTT**				
Hand WDT	0.27	<0.0001	0.15	0.003
Hand CDT	−0.14	0.006	−0.14	0.006
Foot WDT	0.26	<0.0001	0.26	<0.0001
Foot CDT	−0.30	<0.0001	−0.31	<0.0001

r:correlation coefficient. TCNS, Toronto Clinical Neuropathy Score; QTT, quantitative thermal testing; CDT, cold detection threshold; WDT, warm detection threshold.

### Cardiometabolic risk factors for DSPN and QTT parameters associated with DSPN

The baseline cardiovascular risk factors for DSPN and QTT parameters associated with DSPN are listed in Table 4. The statistically significant variables in the univariate logistic regression models included age (years) (*p* = 0.001), diabetes duration (years) (*p* < 0.0001), height (cm) (*p* = 0.01), weight (kg) (*p* = 0.004), waist circumference (cm) (*p* < 0.0001), hyperlipidemia (*p* = 0.007), smoking (*p* = 0.025), retinopathy (*p* = 0.001), coronary artery disease (*p* = 0.007), peripheral artery disease (*p* < 0.0001), hypoglycemic episodes (*p* = 0.001), index HbA1c (%) (mmol/mol) (*p* = 0.001), triglycerides (mg/dL) (*p* < 0.0001), HDL (mg/dL) (*p* = 0.003), hand WDT (*p* = 0.001), foot WDT (*p* < 0.0001), and foot CDT (*p* < 0.0001). These significant univariates were entered into stepwise logistic regression models, and only age (years) (*p* = 0.045), diabetes duration (years) (*p* < 0.0001), waist circumference (cm) (*p* = 0.007), underlying conditions with hypertension and peripheral artery diseases (*p* = 0.004), triglycerides (mg/dL) (*p* = 0.001), and foot CDT (*p* < 0.0001) were independently associated with the presence of DSPN. A decrease of 1°C in the foot CDT increased the DSPN rate by 19%.

**TABLE 4:** Baseline cardiovascular risk factors and QTT parameters associated with DSPN.

	Univariate logistic regression		*E*-value	Multivariate logistic regression	
	OR (95% CI)	Crude *P*-value		Adjusted OR (95% CI)	Adjusted *P*-value
**Characteristics**					
Age (years)	1.05 (1.02–1.09)	0.001	1.28	1.04 (1.0–1.09)	0.045
Sex (male/female)	1.38 (0.85–2.23)	0.19	1		
Diabetes duration (years)	1.09 (1.05–1.12)	<0.0001	1.4	1.08 (1.05–1.13)	<0.0001
Body height (cm^2^)	1.04 (1.0–1.07)	0.01	1		
Body weight (kg)	1.04 (1.0–1.04)	0.004	1		
Waist circumference (cm)	1.04 (1.02–1.07)	<0.0001	1.24	1.04 (1.01–1.07)	0.007
Body mass index	1.05 (0.99–1.1)	0.06	1		
SBP (mmHg)	1.01 (0.99–1.02)	0.25	1		
DBP (mmHg)	1.0 (0.98–1.02)	0.72	1		
Smoking	2.01 (1.10–3.98)	0.025	3.43		
**Underlying disease**					
Hypertension	1.94 (1.05–3.56)	0.03	3.29	2.43 (1.09–5.43)	0.03
Hyperlipidemia	1.93 (1.20–3.12)	0.007	3.27		
Retinopathy	2.52 (1.49–4.24)	0.001	4.48		
Stroke	1.55 (0.89–2.71)	0.12	1		
Coronary artery diseases	3.0 (1.35–6.68)	0.007	5.45		
Peripheral artery diseases	21.64 (4.70–99.68)	<0.0001	42.77	6.1 (1.08–34.31)	0.04
Hypoglycemic episodes	3.48 (1.69–7.16)	0.001	6.42		
**Baseline laboratory test findings**					
Total cholesterol (mg/dL)	1.0 (0.99–1.01)	0.4	1		
Triglycerides (mg/dL)	1.01 (1.0–1.01)	<0.0001	1	1.0 (1.0–1.01)	0.001
HDL (mg/dL)	0.97 (0.95–0.99)	0.003	1.21		
LDL (mg/dL)	1.0 (1.0–1.01)	0.19	1		
UA (mg/dL)	1.09 (0.94–1.26)	0.27	1		
Index HbA1c (%)	2.0 (1.54–2.60)	0.001	3.41		
UACR (mg/mg)	1.0 (0.99–1.0)	0.97	1		
eGFR (mL/min/1.73 m^2^)	1.0 (0.99–1.01)	0.99	1		
**Thermal QTT**					
Hand WDT	1.17 (1.06–1.30)	0.001	1.62		
Hand CDT	0.93 (0.80–1.09)	0.37	1		
Foot WDT	1.12 (1.06–1.20)	<0.0001	1.49		
Foot CDT	0.81 (0.75–0.88)	<0.0001	1.77	0.81 (0.73–0.89)	<0.0001

Data are presented as the OR (95% CI) or as the adjusted OR (95% CI). DSPN, diabetic sensorimotor polyneuropathy; OR, odds ratio; CI, confidence interval; SBP, systolic blood pressure; DBP, diastolic blood pressure; UACR, urine albumin-creatinine ratio; HDL, high-density lipoprotein; LDL, low-density lipoprotein; UA, uric acid; HbA1c, glycated hemoglobin; eGFR, glomerular filtration rate, QTT, quantitative thermal testing, CDT, cold detection threshold; WDT, warm detection threshold.

### Sensitivity analysis using E-value

In the logistic regression model, we use combined cardiovascular risk factors instead of a single risk factor and showed the odds ratio of developing DSPN is 1.35 (1.09–1.67, *p* = 0.006) for one additional risk factor. The result is like a regression model on each risk factor.

To identify the impact of unmeasured confounders such as unmeasured random variability, we calculated the E-value of each risk factor. The E-value in most of our study’s significant risk factor for DSPN is above 3.0, and it tells us that an unmeasured confounder is associated with both treatment and outcome, should be a risk ratio of 3.0-fold more to shift the confidence interval to be the true null hypothesis. Regarding the well-known risk factors such as age and diabetes duration, we standardized age and diabetes duration with z-score and showed an odds ratio of 1.56 (1.2–2.02) and 1.95 (1.55–2.46), respectively. The E-value on foot WDT/CDT value with z-score standardization is 2.65 and 3.12, respectively.

### Serial changes in composite scores of the nerve conduction and parameters of QTT during the study period

After at least 1 year of follow-up, 353 patients completed the follow-up studies, while the other 48 did not. Table 5 shows that the composite scores of the nerve conduction and QTT were stratified by DSPN status from baseline to 1-year follow-up. Regarding the composite scores of the nerve conduction, and QTT parameters at baseline and follow-up in those patients who progressed to the DSPN group, the composite scores of the nerve conduction were significantly higher at the 1-year follow-up (*p* = 0.001), while the hand WDT, hand CDT, foot WDT, and foot CDT did not demonstrate statistically significant differences (*p* = 0.09, *p* = 0.47, *p* = 0.26, and *p* = 0.52, respectively). Concerning the composite scores of the nerve conduction, and QTT parameters at baseline and follow-up in the patients in the regression to no DSPN group, the composite scores of the nerve conduction were significantly lower over 1-year follow-up (*p* < 0.0001), while the hand WDT, hand CDT, foot WDT, and foot CDT did not demonstrate statistically significant differences (*p* = 0.05, *p* = 0.63, *p* = 0.73, and *p* = 0.60, respectively). Furthermore, the composite scores of the nerve conduction, hand WDT, hand CDT, foot WDT, and foot CDT were all statistically significant in the four different DSPN status groups between baseline and the follow-up (*p* < 0.0001, *p* < 0.0001, *p* = 0.001, *p* = 0.002, and *p* < 0.0001, respectively).

**TABLE 5:** Composite scores of the nerve conduction and QTT stratified by the DSPN status at baseline and at 1-year follow-up.

	DSPN status over at the 1-year follow-up				*P*-value^§^
	Remained no DSPN (*n* = 261)	Remained DSPN (*n* = 43)	Regressed to no DSPN (*n* = 29)	Progressed to DSPN (*n* = 20)	
Composite scores					
Baseline	1.5 ± 1.4	6.6 ± 1.8	5.0 ± 2.2^†^	3.6 ± 2.2^†^	<0.0001
Follow-up	1.7 ± 1.6	7.2 ± 1.6	3.6 ± 1.8	5.8 ± 2.2	
**QTT**					
**Hand WDT**					
Baseline	35.1 ± 1.7	36.6 ± 3.4	35.3 ± 1.4	35.5 ± 1.8	<0.0001
Follow-up	34.4 ± 1.2	36.5 ± 3.9	35.9 ± 3.1	35.8 ± 2.3	
**Hand CDT**					
Baseline	29.9 ± 1.4	29.3 ± 1.8	30.0 ± 0.8	29.2 ± 1.4	0.001
Follow-up	30.1 ± 1.2	29.4 ± 2.3	29.8 ± 1.5	28.5 ± 2.1	
**Foot WDT**					
Baseline	40.9 ± 3.9	43.4 ± 3.9	42.3 ± 3.5	40.6 ± 4.6	0.002
Follow-up	39.8 ± 3.7	42.3 ± 3.6	39.7 ± 4.6	41.2 ± 4.8	
**Foot CDT**					
Baseline	28.4 ± 2.2	25.3 ± 3.9	28.2 ± 2.7	27.6 ± 3.1	<0.0001
Follow-up	28.4 ± 2.9	25.8 ± 3.7	28.7 ± 3.2	26.8 ± 4.1	

Data are presented as the mean ± standard deviation. ^§^ Comparison of the parameters among four DSPN status groups between two different stages using repeated-measures ANOVA. ^†^Comparison of each parameter between baseline and follow-up using a paired *t*-test and using *p* < 0.05 to indicate statistical significance. *n*, number of cases; DSPN, diabetic sensorimotor polyneuropathy; QTT, quantitative thermal testing; CDT, cold detection threshold; WDT, warm detection threshold; ANOVA, analysis of variance.

## Discussion

### Major findings of our study

Our study yielded three major findings. First, both the WDT and CDT of the feet were more significantly different between the patients with DSPN and those without DSPN than were the WDT and CDT of the hands. These findings are consistent with the length-dependent nature of diseases in DSPN (Tesfaye, 2011). Second, in addition to cardiometabolic risk factors and microvascular and/or macrovascular complications, the foot CDT is the most promising surrogate biomarker for alternative screening methods for DSPN. Third, we observed a close relationship between the clinical neuropathy scores and large- and small-fiber damage. These findings reflect a predominance of mixed-fiber pathology in DSPN. This may imply a mixed-fiber pathology in DSPN (Itani et al., 2021). Finally, the mixed nerve-fiber damage of DSPN status over a 1-year follow-up was a progressive and reversible change exhibiting a parallel trend between the large- and small-nerve functions.

### Cardiometabolic risk factors for DSPN in T2DM and prediabetes

Prospective population-based studies are useful for ascertaining the frequency of, the severity of, and risk factors for DSPN complications (Tesfaye and Selvarajah, 2009; Tesfaye et al., 2010). However, the identified risk factors may provide important clues to the etiology (Tesfaye et al., 2005). The probability of a risk factor being implicated in the pathophysiology of a complication increases when the same risk factor is consistently observed in different studies. Common risk factors for DSPN are the duration of diabetes and age. The identification of other modifiable potential risk factors, apart from the glycemic control of DSPN, is also important. Several traditional markers for cardiovascular risk have been associated with the development of DSPN, including smoking, obesity, triglycerides, and hypertension, which are independent risk factors (Tesfaye and Selvarajah, 2012). Further clinical trials are needed to confirm the contributions of modifiable cardiovascular risk factors and the outcomes of guiding the treatment plan for DSPN (Tesfaye and Selvarajah, 2009), and the present study demonstrated that these risk factors contributed to DSPN in patients with T2DM and patients with prediabetes.

### QTT for the early diagnosis and follow-up of DSPN

The validity, reproducibility, and time-saving diagnostic techniques available for an early diagnosis and follow-up of patients with DSPN in clinical practice remain unsatisfactory. Currently, DSPN is diagnosed primarily based on interviews and the physical examination of characteristic symptoms and signs (Dyck et al., 2011a). NCS has the characteristics of being quantifiable and objective. The face is not surprising since both large and small neuropathies are frequently combined (Itani et al., 2021). The QTT can be used for non-invasive and time-saving assessments of sensory nerve function. Clinical studies have demonstrated that the QTT can identify 93% of patients with impaired glucose tolerance or T2DM (Vlckova-Moravcova et al., 2008) and one-half of asymptomatic participants with T2DM with a normal NCS (Jimenez-Cohl et al., 2012). The QTT can also be valid for monitoring the progression of the disease at follow-up, which has been demonstrated in previous studies (Dyck et al., 1978; Shy et al., 2003; Chong and Cros, 2004) and in our study. The prospective 5-year longitudinal study showed particularly neuropathic deficits, intraepidermal nerve fiber density (IENFD), and NCS could produce clinically meaningful degrees of progression and regression (Ziegler et al., 2021). These findings highlight appropriate endpoints that could find favorable factors that influence DSPN. Although small fiber can pathologically be evidenced by reduced IENFD being prominent at baseline, small fiber dysfunction was determined only to a minor extent, while large fiber dysfunction was detected by the NCS, and was considerably more frequent and closer to the prevalence of abnormal IENFD. These findings were consistent with our study. Our study also demonstrated that the DSPN status over a 1-year follow-up was a progressive and reversible change in each QTT parameter. This makes sense because DSPN is the total number of symptoms and functional alterations of various classes of fibers.

### Foot CDT as a surrogate electrophysiologic marker for DSPN

Regarding the small-nerve fiber function, warm sensation and heat pain are attributed to C fibers, the cold sensation is attributed to A-δ fibers, and cold pain is attributed to a mix of C and A-δ fibers (Morin and Bushnell, 1998). Previously published studies demonstrated A-δ fiber-mediated cold threshold determination is most often impaired (Hilz et al., 1988; Loseth et al., 2008; Malik, 2008), and it is easier to detect cooling more precisely than warming (Loseth et al., 2008). Loseth et al. (2008) also showed that patients with diabetes and normal nerve conduction studies had significantly higher CDT. The foot CDT was selected as the most promising surrogate marker strongly associated with the presence of DSPN, which reflects the length-dependent pattern of DSPN (Papanas and Ziegler, 2015) and CDT as a more sensitive parameter than the WDT (Hilz et al., 1988; Loseth et al., 2008). QTT is known to be highly subjective. The DSPN reversibility and progression also be influenced by within-person variability. To obtain accuracy and agreement in the assessment of neuropathic signs, the neuromuscular experts agree to scoring only in unequivocal abnormality and use the percentile to grade the severity (Dyck et al., 2017). Likewise, to get proficient QST results, highly standardized stimuli, algorithms of testing, and finding threshold and reference values should be used (Dyck et al., 2017). To accurately assess the attributes of nerve conduction study, highly standardized and referenced methods also should be used (Dyck et al., 2017).

Besides the clinical scale, composite scores of representative attributes of nerve conduction are useful in estimating the severity of polyneuropathies in the Rochester Diabetic Neuropathy Study (England et al., 2009; Dyck et al., 2011a). Recently, two completed phase III trials have developed the measurement of the severity of Transthyretin Familial Amyloid Polyneuropathy (TTR-FAP) (Adams et al., 2017; Dyck et al., 2017). Two major changes from the previous Neuropathy Impairment Score + 7 tests (NIS + 7) were revised in nerve conduction studies (NCS). First, they modified the scoring system of NCS into a composite amplitude score (CAS) (Suanprasert et al., 2014) because conduction velocity and distal latency measurements are often unmeasurable in severe amyloid polyneuropathy (Dyck et al., 2011b). Second, they added upper limb amplitudes of motor and sensory nerve conductions to achieve a length-dependent distribution of the neuropathy. This change in NCS measurement markedly improved the correlation of mNIS + 7 with overall NIS in TTR-FAP. In this study, we adapted CAS from our recent study as the measurement of the severity of nerve conduction in DSPN (Lai et al., 2019) because both TTR-FAP and DSPN are mainly axonal pathophysiology (Lai et al., 2020). Except for the differences in ulnar and sural sensory nerve conduction velocity (SNCV), the remaining NCS studies between TTR-FAP and DSPN groups were not different (Escolano-Lozano et al., 2018).

### Study limitations

This study had several limitations. First, the QTT requires participant cooperation and understanding of the instructions, and the subjective nature of the testing and a lack of consensus on standard practice further limit the QTT (Shy et al., 2003). Second, it cannot differentiate lesions originating from the central nervous system from those originating from the peripheral nervous system and can be used under the clinical characteristic symptoms and signs of DSPN. Finally, normative QTT data were not available for use as a reference for patients older than 80 years (Yarnitsky and Sprecher, 1994) in other studies and our study, which may limit its use in the elderly patient group.

## Conclusion

We observed a close relationship between the clinical neuropathy scores and large and small fiber damage. These findings reflect mixed-fiber pathologies in DSPN. The DSPN status over a 1-year follow-up was a progressive and reversible change with a parallel trend between large and small nerve functions. These findings summarize and show the feasibility of QTT as a time-saving screening and follow-up service in patients with clinically suspected DSPN. Concerning the parameters in QTT, we also identified that the foot CDT is the most promising surrogate biomarker for alternative DSPN screening methods.

## Data availability statement

The raw data supporting the conclusions of this article will be made available by the authors, without undue reservation.

## Ethics statement

This study conformed to the guidelines of the Declaration of Helsinki, and it has been approved by the Institutional Review Board of Chang Gung Memorial Hospital (201901363B0 and 202002095B0). The patients/participants provided their written informed consent to participate in this study.

## Author contributions

Y-RL performed the statistical analysis, conceived the study, participated in its design and coordination, and helped draft the manuscript. Y-CH, Y-CC, W-CC, C-CH, B-CC, C-EK, T-YL, and H-CC participated in the sequence alignment and clinical evaluation of patients. All authors read and approved the final manuscript.
